# Two new species and a new record of yellow *Cantharellus* from tropical *Quercus* forests in eastern Mexico with the proposal of a new name for the replacement of *Craterellus confluens*

**DOI:** 10.3897/mycokeys.80.61443

**Published:** 2021-05-20

**Authors:** Leticia Montoya, Mariana Herrera, Victor M. Bandala, Antero Ramos

**Affiliations:** 1 Red Biodiversidad y Sistemática, Instituto de Ecología A.C., P.O. Box 63, Xalapa, Veracruz, 91000, México

**Keywords:** American *Cantharellus*, *
Craterellus
*, ectomycorrhizal mushrooms, Neotropical *Cantharellus* or chanterelles, oak, wild edible mushrooms

## Abstract

Two new species of yellow *Cantharellus* and a new record of *Cantharellus tabernensis* associated with tropical species of *Quercus* are presented, based on the taxonomic study of fresh specimens and in a phylogenetic analysis of transcription elongation factor 1-alpha (*tef*-1α) and the large subunit of the ribosome (nLSU) sequences. One of the new species proposed here, corresponds to a choice edible mushroom, which, in our molecular phylogeny, resulted in it being related to the group of species around *C. lateritius* and sister with *Craterellus confluens* type specimen. This latter is here formally transferred to *Cantharellus* and consequently a new name, *Cantharellus furcatus*, is proposed to replace the homonym *Cantharellus confluens* (Schwein.) Schwein. 1834 a later synonym of *Byssomerulius corium*. Detailed macroscopic and microscopic descriptions accompanied with illustrations and a taxonomic discussion are presented for each species.

## Introduction

In the American continent, especially from USA, new species of *Cantharellus* had been proposed, several of them look-alikes of the commonly cited *C. cibarius* Fr., *C. cinnabarinus* Fr. and *C. lateritius* (Berk.) Singer (Arora and Dunham 2008; Buyck et al. 2010, 2011, 2016a, b; Buyck and Hofstetter 2011; Foltz et al. 2013; Leacock et al. 2016; Thorn et al. 2017). Further explorations in tropical America are achieving also the discovery of undescribed species of the genus (e.g. Wartchow et al. 2012; Henkel et al. 2014; Nasc﻿imento et al. 2014; Buyck et al. 2016b; Herrera et al. 2018) as occurred also with *Craterellus* (Wilson et al. 2012).

Species delimitation in *Cantharellus* is often said to be hard to address, especially because of the overlap of phenotypic variation, including scarce microscopic morpho-anatomic taxonomically informative features. In such a sense, Buyck et al. (2014) explicitly defined that basidiomes of *Cantharellus* species “…under the microscope …exhibit a discouraging monotony…”. Studying *Cantharellus* specimens from Mexico, we have noted that the difficulty in revising early records is exacerbated by frequent incomplete data accompanying herbaria specimens. For or instance, there is poor or no information on features like hymenophore and color variations of basidiomes along their development or even by weathering effects. It is of primary importance then, to be able to count on accurate observations of specimens in fresh that lead to the characterization of their phenotypes and establish robust concepts for pertinent taxonomic conclusions. It is important even to count on data on the spatial/temporal distribution, and associated tree species.

In *Cantharellus violaceovinosus* (Herrera et al. 2018), for example, it was possible to document wide macromorphological and color information through a register of samples collected over more than five years, even in weekly explorations along three years sampling. Such a record allowed us to recognize its phenology between July-October in pure stands of *Quercus oleoides*, and found it less frequent in association with *Q. glaucescens* and *Q. sapotifolia*. In fact, such a record together with molecular information facilitated the distinction of *C. violaceovinosus* from other phenotypically similar species. Olariaga et al. (2015) informed about the identity of some taxa previously described solely based on colored or unpigmented variants, i.e., while in *C. amethysteus* (Quel.) Sacc., *C. cibarius* Fr., *C. ferruginascens* P.D. Orton, *C. pallens* Pilát and *C. romagnesianus* Eyssart. & Buyck, white specimens may occasionally occur, in *C. cibarius* and *C. pallens* orange forms can be found. Among other conclusions, these authors demonstrated that white forms of *C. cibarius* already described as varieties (var. inodorus Velen, f. pallidus R. Schulz) corresponded molecularly indeed to a single taxon, and *C. gallaecicus* (Blanco-Dios) Olariaga, lacking yellow-orange tones, is in fact the same as the orange-yellow to ochre-yellow *C. romagnesianus* (Olariaga et al. 2017).

Yellow chantherelles, such as *Cantharellus cibarius* Fr., *C. lateritius* (Berk.) Singer, *C. odoratus* (Schwein.) Fr. and *Craterellus confluens* Berk. & M.A. Curtis have been reported from different regions of Mexico (Berkeley 1867; Guzmán and Sampieri 1984; Guzmán 1985; Guevara et al. 2004; Pérez-Moreno et al. 2008; Garibay-Orijel et al. 2009; Kong et al. 2018; Corona-González 2019). *Craterellus confluens* was described by Berkeley (1867) from Mexico (Orizaba at central Veracruz state), in a locality relatively close to one of the current study sites in the Municipality of Zentla, Veracruz. Particularly from this latter region, an edible yellow chantherelle species common in the surrounding *Quercus* forest and even sold in popular markets, was previously reported under *C. odoratus*, considering it contaxic with *Cr. confluens* (Guzmán and Sampieri 1984; Guzmán 1985).

During a systematic multiyear sampling of basidiomes, as part of a project focused to study ectomycorrhizal fungi in tropical *Quercus* forests in eastern Mexico (Montoya et al. 2019a, b), we found coexisting three species of yellow *Cantharellus*. Two of these taxa are distinctive by having short-sized basidiomes with veined to gill-like folded hymenophore, while a third one, is distinctive by its medium-sized, moderately robust basidiomes, with smooth or at times rugulose hymenophore, this latter apparently corresponding to what was earlier reported as “*C. odoratus*”.

We report here the results of both, a morphological study of fresh specimens and a phylogenetic analysis of the transcription elongation factor 1-alpha (*tef*-1α) and the large subunit of the ribosome (nLSU) sequences obtained from our recent collections and those available in GeneBank. Three well-supported clades inferred in the phylogenetic tree, allowed us to recognize two new species and the record in Mexico of *C. tabernensis*, described from Southern Mississippi in USA (Feibelman et al. 1996). One of the new species here proposed, corresponds to the yellow *Cantharellus* with smooth hymenophore, which interestingly, in our phylogenetic analysis appears independent of *Craterellus confluens* (holotype), *Cantharellus lateritius* (holotype) and *C. flavolateritius* Buyck & V. Hofst. (paratype) sequences. Both macromorphological and color variation mentioned in the descriptions were recovered from fresh basidiomes through seven years of sampling. The monitoring of monodominant stands of three different species of tropical *Quercus*, allowed registering also, the putative ectomycorrhizal interaction of the studied species of *Cantharellus*.

## Materials and methods

### Sampling and morphological study

*Cantharellus* basidiomes were collected through a weekly sampling during June-October 2015–2018, with sporadic collections among 2009–2014, in tropical oak forests from Municipalities of both Zentla (837–850 m a.s.l.) and Alto Lucero (400–500 m a.s.l.) in central Veracruz (eastern Mexico). In these oak forests, *Quercus oleoides* is dominant, and even forming pure stands. In the Zentla locality, *Q. glaucescens* and *Q. sapotifolia* are also present, and form monodominant small stands. Descriptions are derived from recording the morpho-anatomical features of fresh samples, the records of color follow Kornerup and Wanscher (1978) (e.g. 4A4–8) and Munsell (1994) (e.g. 2.5Y 7/8–8/8). Basidiomes were dried in a hot air dehydrator (45 °C) for their preservation. Microscopic features were examined from desiccated specimens, measured in 3% KOH and stained with 1% Congo red or analyzed in Melzer´s solution. Thirty-five basidiospores per collection were measured in lateral view following Montoya et al. (2019b). In the descriptions *X*– denotes an interval of mean values of basidiospores length and width per collection in *n* collections, and *Q*– refers to the range of coef. Q (where Q is the average of the ratio of basidiospore length/basidiospore width in each collection). Line drawings were made with the aid of a drawing tube. Collections form part of XAL Herbarium (Thiers B. [continuously updated] Index Herbariorum: a global directory of public herbaria and associate staff. New York Botanical Garden`s Virtual Herbarium. http://sweetgum.nybg.org/science/ih/).

### DNA extraction, PCR and sequencing

Genomic DNA was isolated from fresh material according to Cesar et al. (2018). We amplified the transcription elongation factor 1-alpha (*tef*-1α) using the pairs of primers *tef*-1F/*tef*-1R (Morehouse et al. 2003) and *tef*-1Fcanth/*tef*-1Rcanth (Buyck et al. 2014). We amplified the large subunit of ribosome (nLSU) using combinations of the pair of primers LR0R/LR7 (Vilgalys and Hesler 1990) and the pair of primers designed LRCA1(5'-GTTGCACTGTCCGAGTTGTA-3')/LRCA2(5'-AGACTGATGGCGAGGTATGA-3'). PCR was performed according to Herrera et al. (2018). A capillary sequencer, Genetic Analyzer 3730XL (Applied Biosystems), was used to obtain the sequences of the amplified PCR products. These sequences were assembled, edited, and deposited at GenBank database (Benson et al. 2017), the accession numbers are indicated in Table 1.

**Table 1.** *Cantharellus* taxa: Fungal names, specimen vouchers, locations and GenBank accession numbers (for 28S and tef-1α). Newly sequenced collections in bold.

Taxa	Voucher	Locality	LSU	*tef–1α*
*Cantharellus addaiensis*	BB 98.033 TYPE	Tanzania	KF294667	JX192992
*Cantharellus afrocibarius*	BB 96.235 TYPE	Zambia	KF294668	JX192993
*Cantharellus albidolutescens*	BB 08.070 TYPE	Madagascar	KF294646	JX192982
*Cantharellus alborufescens*	BB 12.075	Italy	KX929161	KX907243
	BB 12.076	Italy	KX907222	KX907244
*Cantharellus altipes*	BB 07.019 TYPE	USA	KF294627	GQ914939
*Cantharellus ambohitantelyensis*	BB 08.336 TYPE	Madagascar	KF294656	JX192989
*Cantharellus amethysteus*	AH44796 TYPE	Spain	KR677550	KX828819
*Cantharellus appalachiensis*	BB 07.123	USA	KF294635	GQ914979
*Cantharellus camphoratus*	TENN:F-38025 TYPE	Canada	KX896788	–
*Cantharellus cerinoalbus*	AV 06.051 TYPE	Malaysia	KF294663	–
*Cantharellus cibarius*	BIO10986 TYPE	Sweden	KR677539	KX828823
*Cantharellus cinnabarinus*	BB 07.001 TYPE	USA	KF294624	GQ914985
*Cantharellus coccolobae*	1065/RC 11.25 TYPE	Guadeloupe	KX857089	KX857021
*Cantharellus congolensis*	BB 98.039	Tanzania	KF294609	JX193015
	BB 98.058	Tanzania	KF294673	JX192996
*Cantharellus corallinus*	JJ MO-Canth-2 TYPE	USA	KX896776	KX857031
*Cantharellus decolorans*	BB 08.278 TYPE	Madagascar	KF294654	GQ914968
*Cantharellus enelensis*	13.08.21.av02 TYPE	Canada	KX592712	–
*Cantharellus ferruginascens*	BB 07.283	Slovakia	KF294638	GQ914952
*Cantharellus fistulosus*	DT 43 TYPE	Tanzania	KF294674	JX192992
***Cantharellus flavolateritius***	**Halling 6252**	**USA**	**MT371334**	–
	JJ/NC-CANT-2	USA	KX896783	KX857027
*Cantharellus flavus*	C066WI TYPE	USA	JX030437	–
*Cantharellus formosus*	SAR220712	Canada	KR677553	KX828830
*Cantharellus friesii*	AH44798	Spain	KR677522	KX828831
*Cantharellus garnierii*	RF32 PC TYPE	New Caledonia	AY392767	–
*Cantharellus gracilis*	BB 98.234 TYPE	Tanzania	KF294612	JX192970
*Cantharellus guyanensis*	1501/MRG07	Guyane	KX857094	KX857060
	1517/MR	Guyane	KX857095	KX857061
*Cantharellus hainanensis*	N.K. Zeng2289 TYPE	China	KY407524	KY407536
*Cantharellus heinemannianus*	BB 96.307 TYPE	Zambia	KF294665	–
*Cantharellus humidicolus*	BB 98.036 TYPE	Tanzania	KF294666	JX193005
*Cantharellus ibityensis*	BB 08.196 TYPE	Madagascar	KF294650	GQ914980
Cantharellus isabellinus var. parvisporus	BB 98.020 TYPE	Tanzania	KF294614	JX192972
*Cantharellus iuventateviridis*	BP Looney 523 TYPE	USA	NG_060428	KX857047
***Cantharellus lateritius***	**TJ Baroni 8059F**	**USA**	**MT371335**	–
	**TJ Baroni 8117L**	**USA**	**MT371336**	–
	BB 07.025 TYPE	USA	KF294633	GQ914959
*Cantharellus lewisii*	BB 07.003 TYPE	USA	JN940597	GQ914962
*Cantharellus lilacinopruinatus*	BB 07.221	Slovakia	KF294637	GQ914951
*Cantharellus miniatescens*	1683/TH9870	Cameroon	KX857108	KX857079
*Cantharellus minor*	BB 07.002	USA	KF294625	JX192978
	BB 07.057	USA	KF294632	JX192979
*Cantharellus miomboensis*	BB 98.021 TYPE	Tanzania	KF294613	JX192971
*Cantharellus pallens*	BB 09.441	Italy	KX907218	KX907240
	BB 12.082	Italy	KX857092	KX857036
***Cantharellus parvoflavus***	**Montoya 5423 TYPE**	**Mexico**	**MT371337**	**MT449706**
	**Herrera 204**	**Mexico**	**MT371338**	**MT449707**
	**Herrera 229**	**Mexico**	**MT371339**	**MT449708**
*Cantharellus paucifurcatus*	BB 08.320 TYPE	Madagascar	KF294655	JX192988
*Cantharellus phasmatis*	C073WI TYPE	USA	JX030426	–
*Cantharellus platyphyllus*	BB 98.126 TYPE	Tanzania	KF294620	JX192975
Cantharellus platyphyllus subsp. bojeriensis	BB 08.160	Madagascar	KF294648	JX192984
*Cantharellus pseudominimus*	JV 00.663	France	KF294657	JX192991
*Cantharellus quercophilus*	BB 07.097 TYPE	USA	KF294644	JX192981
*Cantharellus romagnesianus*	AH44218	Spain	KX828807	KX828836
*Cantharellus roseofagetorum*	AH44789 TYPE	Georgia	NG_058962	KX828839
*Cantharellus sebosus*	BB 08.234 TYPE	Madagascar	KF294652	JX192986
*Cantharellus spectaculus*	C081WI TYPE	USA	JX030421	JX030414
*Cantharellus splendens*	BB 96.306 TYPE	Zambia	KF294670	–
*Cantharellus subalbidus*	BB 13.014B	USA	KX896782	KX857038
*Cantharellus subamethysteus*	DS 06.218 TYPE	Malaysia	KF294664	–
*Cantharellus subcyanoxanthus*	BB 00.1137 TYPE	Madagascar	–	JX192990
Cantharellus subincarnatus subsp. rubrosalmoneus	BB 06.080 TYPE	Madagascar	KF294601	JX192962
*Cantharellus symoensii*	BB 98.113 TYPE	Tanzania	KF294619	JX192974
***Cantharellus tabernensis***	**Herrera 120**	**Mexico**	**MT371340**	**MT449709**
	**Herrera 121**	**Mexico**	**MT371341**	**MT449710**
	BB 07.056 TYPE	USA	KF294631	GQ914974
*Cantharellus tanzanicus*	BB 98.040 TYPE	Tanzania	KF294622	JX192977
*Cantharellus tenuithrix*	BB 07.125 TYPE	USA	JN940600	GQ914947
*Cantharellus texensis*	BB 07.018 TYPE	USA	KF294626	GQ914988
*Cantharellus tomentosus*	BB 98.060 TYPE	Tanzania	KF294672	JX192995
***Cantharellus veraecrucis***	**Herrera 142**	**Mexico**	**MT371342**	–
	**Herrera 58**	**Mexico**	**MT371343**	**MT449711**
	**Bandala 4505 TYPE**	**Mexico**	**MT371344**	**MT449712**
*Cantharellus violaceovinosus*	Corona 648 TYPE	Mexico	NG_064465	MF616521
***Craterellus confluens***	**Botteri 6 TYPE**	**Mexico**	**MT371345**	–
*Craterellus tubaeformis*	BB 07.293	Slovakia	KF294640	GQ914989
*Hydnum repandum*	BB 07.341	Slovakia	KF294643	JX192980

### Phylogenetic analysis

**Figure 1.**
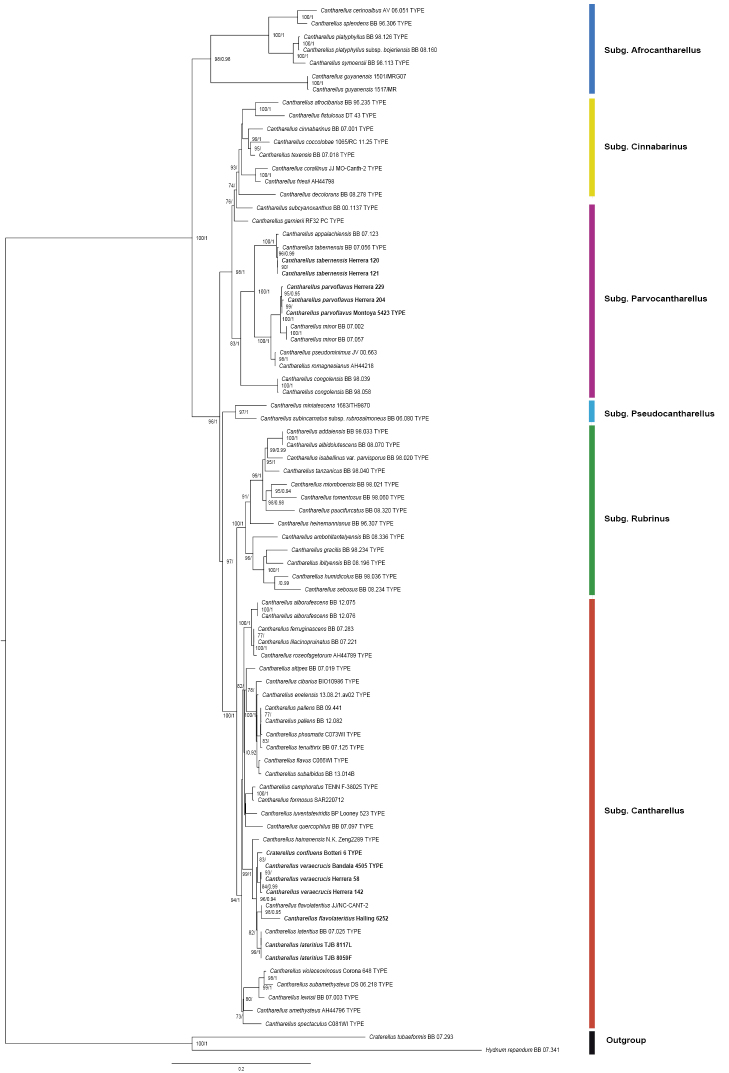
Phylogenetic relationships within *Cantharellus* species inferred from the combined nLSU (large subunit of the ribosome) and *tef-1α* (transcription elongation factor 1-alpha) sequences, by maximum likelihood method and Bayesian inference. The new species are indicated in bold letters. Bootstrap scores (only values ≥ 70) and Bayesian Posterior Probabilities (only values ≥ 0.90) are indicated above branches.

We constructed a concatenated dataset, using PhyDE v.0.9971 (Müller et al. 2010), with 19 sequences obtained here (nLSU and *tef*-1α) (Table 1), together with sequences of related taxonomic groups, and additionally taking as reference works on chantarelles by An et al. (2017), Buyck et al. (2014, 2016a, b, c, d), Herrera et al. (2018) and Olariaga et al. (2017). The dataset was aligned with MAFFT online service (Katoh et al. 2019), and the inconsistencies were corrected manually. Phylogenetic trees were generated according to Montoya et al. (2019a). The evolutionary model was calculated using the IQ-Tree 2.0-rc1 (Minh et al. 2020; Kalyaanamoorthy et al. 2017) and the best-fit model selected using the Bayesian Information Criterion (BIC), the Akaike Information Criterion (AIC) and corrected AIC. This later was used to generate a phylogenetic tree with the Maximum Likelihood (ML) method, with a Nearest Neighbour Interchange (NNI) heuristic, with the TIMe+I+G4 evolutionary model. A consensus tree was also generated calculating the Robinson-Foulds distance between the ML tree and the consensus tree, the branches being tested by means of Ultrafast Approach Bootstrap (UFBoot), SH-like approximate Likelihood Ratio Test (SH-aLRT), Approximate Bayes test (aBayes) and Bootstrap Standard (BS). Another phylogenetic tree (not shown) was also generated by Bayesian Inference (BI), using Mr Bayes v. 3.2.7 (Ronquist et al. 2012) according to Montoya et al. (2019a), with the previously calculated evolutionary model. The phylogenies from ML and BI analyses were displayed using FigTree v1.4.4 (Rambaut 2018). Only bootstrap values (BS) of ≥ 70% and Bayesian posterior probabilities (BPP) of ≥ 0.90 were considered and indicated on the tree branches (BS/BPP) of Fig. 1.

## Results

We studied 78 specimens in the field (not all conserved) of *Cantharellus* species, each with basidiomes in different growth stages, most of them showing an annual fruiting pattern between August-October. We generated 19 new DNA sequences from eight fresh specimens and four from desiccated herbarium collections, twelve from nLSU and seven from *tef*-1α (Table 1). The built dataset includes a total of 80 sequences, using *Craterellus tubaeformis* and *Hydnum repandum* sequences as the outgroup (Table 1); the alignment is deposited in TreeBASE as 26146. In the inferred molecular phylogeny two groups of the produced sequences clustered in isolated position. One of them, the *Cantharellus* with smooth hymenophore, showed relationships with *Craterellus confluens*, *Cantharellus lateritius* and *C. flavolateritius*, and the other group appeared close to *C. minor* and *C. romagnesianus* (Fig. 1). Based on the distinctive morphological features and color variation of specimens of two clades, as well as their isolated position in the phylogeny obtained, we concluded that these Mexican specimens represent two distinct species, which are proposed here as new to science (described below). A third group of sequences clustered with strong support together with sequences of *C. tabernensis* type specimen (Fig. 1). Although Mexican samples, in contrast with the morphological description by Feibelman et al. (1996) that shows some minor differences (below discussed), all share the taxonomic distinctive characters to interpret them as being conspecific. In the classification proposed by Buyck et al. (2014) the *Cantharellus* with smooth hymenophore, clustered within subgenus Cantharellus and the other new species proposed here, together with *C. tabernensis*, within subgenus Parvocantharellus.

### Taxonomy

#### Description of the new species

##### Cantharellus veraecrucis

Taxon classificationFungiScleractiniaFungiidae

Bandala, Montoya & M. Herrera sp. nov.

03B35EAE-DF06-5FA6-B529-485DFF5EB90C

838105

Figs 2a, b , 3

###### Holotype.

Mexico. Veracruz: Municipality of Zentla, around town of Zentla, 850 m a.s.l., gregarious on ground, under *Quercus oleoides* Schltdl. & Cham., 5 July 2012, Bandala 4505 (XAL).

###### Diagnosis.

Differing from other related yellow Cantharellus species (subgenus Cantharellus) by the smooth hymenophore, often rugulose or with low, close, fine, irregular veins, pinkish-yellow, ellipsoid basidiospores 7–9 (–10.5) × (4.5–) 5–6.5 µm [*Q*–= 1.36–1.65], basidia (43–) 49–96 (–104) × 5–12 µm, pileipellis terminal hyphae 22–60 (–73) × 4–5.5 µm, subcylindrical, rarely subventricose, straight to moderately flexuous, wall ≤ 1 µm thick.

**Figure 2.**
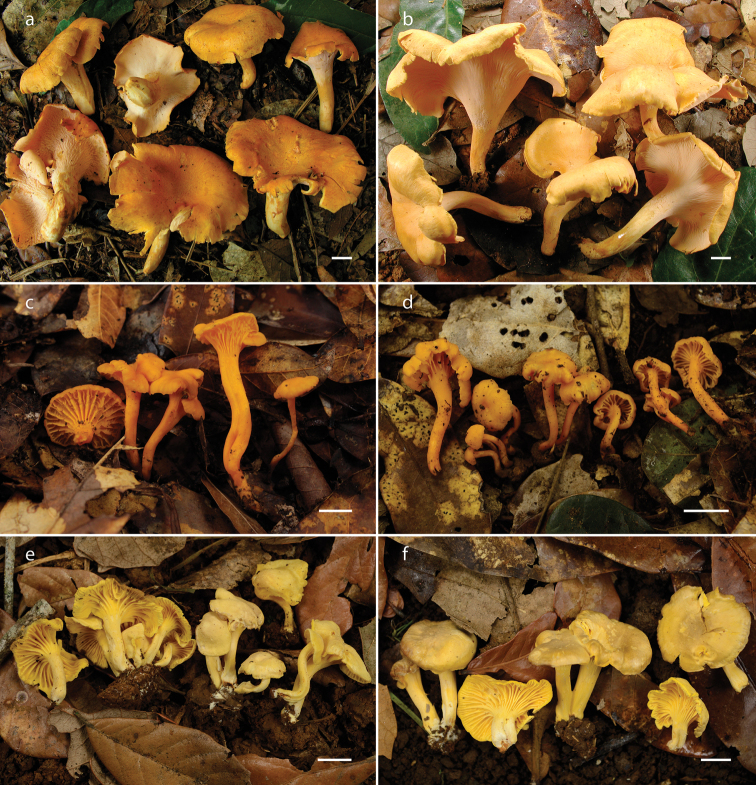
Basidiomes of *Cantharellus* species **a, b***C. veraecrucis* (**a** Bandala 4505, holotype **b** Herrera 142) **c, d***C. parvoflavus* (**c** Montoya 5423, holotype **d** Herrera 229) **e, f***C. tabernensis* (**e** Herrera 120 **f** Herrera 131). Scale bars: 10 mm.

###### Gene sequences ex-holotype.

nLSUMT371344; *tef*-1α MT449712.

###### Etymology.

Referring to the locality of origin, in the State of Veracruz, Mexico.

###### Description.

***Pileus*** 20–80 (–100) mm diam, convex to plane convex, then more or less applanate and centrally depressed, becoming concave and finally broadly infundibuliform; involute margin when young, later incurved and becoming recurved or plane or uplifted in old specimens, not striate, at first entire, becoming variably lobed and undulate; surface dry, when young with appressed fibrils forming a moderately fine, squamulose surface especially at the center, smooth to glabrescent with age, yellow, light yellow (2.5Y 8/3, 7/12, 10YR 4–5/2), pale orange to bright yellow-orange (3A7–8, 4A4–8, 5A4–8, 2.5Y 7/8–8/8, 10YR 6/8, 7/6–8, 8/8) and even brownish-orange (5B7), at times light gray (10YR 7/1–2, 7.5YR 7/1, 4B2) at the center, orange-buff (5B5), salmon-orange to dirty peach-orange (6A6, 6B3, 6B5) or even brown (6E5). ***Hymenophore*** decurrent, smooth overall, often rugulose or with low, close, simple or forked, fine, irregular veins; paler than the pileus, light rose (10YR 8/2–3;7.5YR 7/3–4, 8/4, 5A2–4) when young although with age still preserving pinkish tints on a pale yellow (4A2–3), light yellow (10YR 8/3–4, 8/6, 2.5Y 8/4), light orange (6A3–4), or even egg yellow (4A8) ground. ***Stipe*** 10–75 × 6–21 mm, equal, tapering gradually downwards, somewhat sinuous or curved, central, occasionally somewhat eccentric, solid, glabrous to subtomentose, at times with age the surface becomes detached in scattered fibrils concolorous with hymenophore, whitish with yellow tinges (4A3–4), pale to bright yellow (4A6–8), orange (5A4), to orange-brown tinges (4A8, 4B7–8, 5B7) especially towards the base, often staining ochraceous or rusty orange color when handle; base in some specimens villous to finely villous under lens. ***Context*** fleshy, fibrous in stipe, concolorous with pileus or paler, yellowish-buff, odor agreeable fruity, faintly to peach or somewhat recalling butter; taste mild, fruity agreeable, finally somewhat bitter. KOH 3% negative, only somewhat orange on pileus, NH_4_OH 10% negative.

**Figure 3.**
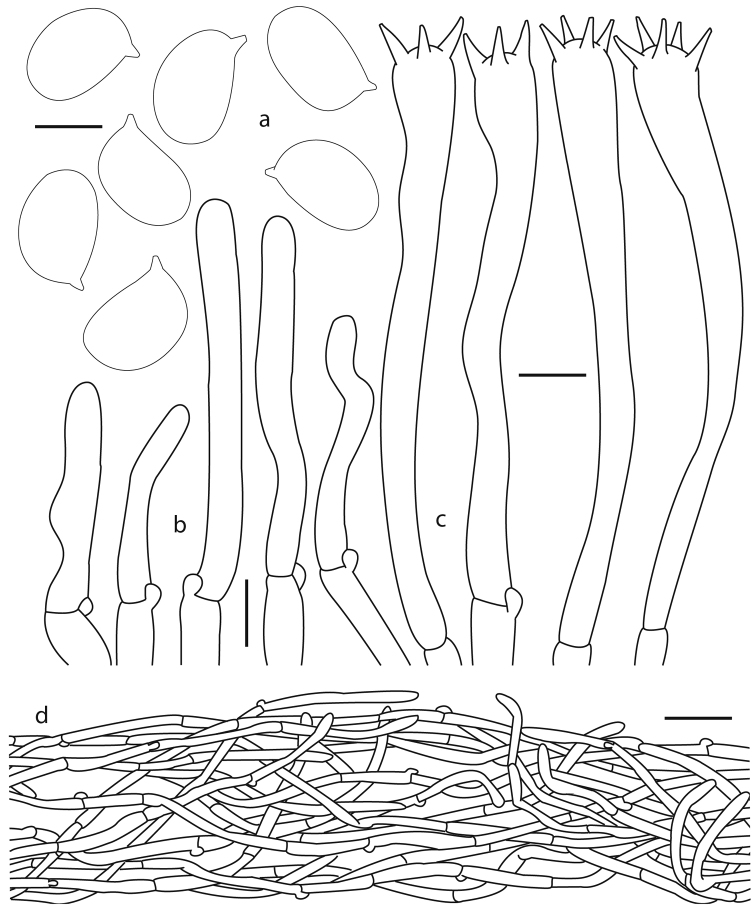
*Cantharellus veraecrucis* (Bandala 4505, holotype) **a** basidiospores **b** terminal elements of the pileipellis **c** basidia **d** longitudinal section of pileipellis. Scale bars: 5 μm (**a**); 10 μm (**b, c**); 25 μm (**d**).

***Basidiospores*** 7–9 (–10.5) × (4.5–) 5–6.5 µm [*X*– = 7.8–8.9 × 5.3–6.1 µm, *Q*– = 1.36–1.65 (n = 12)], ellipsoid, smooth, thin-walled, hyaline, inamyloid. ***Basidia*** (43–) 49–96 (–104) × 5–12 µm, narrowly clavate to subcylindrical, with 2–5 sterigmata, thin-walled, hyaline, subhymenium composed of cylindrical hyphae 3–5 µm diam. ***Cystidia*** absent. ***Pileipellis*** a cutis composed of cylindrical hyphae 4–6 µm diam., intermingled in a compact arrangement, hyaline, yellowish colored in group; terminal hyphae 22–60 (–73) × 4–5.5 µm, subcylindrical, rarely subventricose, scattered, straight to moderately flexuous, smooth, hyaline, inamyloid, thick-walled (<1 µm thick). ***Pileus trama*** composed of cylindrical hyphae, 4–5 µm diam, slightly thick-walled (<1 µm thick), hyaline, some with weakly refringent contents. ***Hymenophoral trama*** composed of hyphae 4–5 µm diam, thin-walled, some with weakly refringent contents. Clamp connections present in all tissues.

###### Habitat.

Solitary to gregarious, on soil, in tropical oak forest, in the studied sites it is recorded frequently in monodominant stands of *Quercus oleoides*, being less frequent in monodominant stands of *Q. glaucescens* Bonpl. or *Q. sapotifolia* Liebm.; fruiting in June-October at the coastal plain of central Veracruz State, east coast of Mexico.

###### Specimens examined.

Mexico. Veracruz, Municipality of Zentla, Road Puentecilla-La Piña, 837 m a.s.l., 2 Jul 2009, Ramos 192, 193, 194; 21 Jun 2012, Herrera 23, 24, 28; 5 Jul 2012, Corona 649, 650, 653; 31 Jul 2012, Montoya 4887; 6 Nov 2013, Herrera 68. Around town of Zentla, 850 m a.s.l., 26 Jun 2013, Herrera 58, 59; 15 Jun 2016, Herrera 153, 154; 23 Jun 2016, Herrera 156; 6 Jul 2016, Herrera 175, 181, 183; 12 Jul 2016, Caro 71, Herrera 185, 186, 188, 190; 10 Aug 2016, Herrera 193; 5 Oct 2016, Melecio 16; 23 Oct 2018, Herrera 159; 12 Jul 2017, Montoya 5347; 21 Sep 2017, Garay 394, Garrido 88, 89; 20 Jun 2018, Herrera 232. Municipality of Alto Lucero, NE Mesa de Venticuatro, 450–500 m a.s.l., 17 Sep 2015, Herrera 140; 4 Sep 2018, Herrera 244. Jaguarundi Park, Coatzacoalcos 29 Sep 2015, Herrera 142, 143, 144, 145 (all at XAL).

###### Remarks.

*Cantharellus veraecrucis* is distinguished by the basidiome colors, hymenophore smooth (or at times discontinuously rugulose) with pinkish tinges, and pileus surface with appressed fibrils. In some stage of development, it superficially might look like *C. flavolateritius*; this latter, however, according to Buyck et al. (2016a) exhibits bright yellow colors on pileus, the hymenophore is composed of radially oriented, low anastomosing veins,“… locally almost smooth…”, paler stipe (yellow to off-white), narrowly ellipsoid, somewhat phaseoliform basidiospores (7.1–) 7.2–7.88–8.5 (–10.0) × (4.0–) 4.2– 4.71–5.2 (–5.8) μm, Q = (1.4–) 1.5–1.69–1.8 (–2.1) and pileipellis terminal hyphae often rather short, clavulate or apically slightly inflated, rarely ellipsoid, mostly 20–50 (–70) μm long, sometimes more or less wavy-undulate in outline.

In our phylogenetic analysis, *C. veraecrucis* is related also with *C. lateritius*. This latter species exhibits pale to deep yellow or even apricot orange (Buyck et al. 2011) or bright orange or slightly pinkish orange colors (Petersen 1979). Buyck et al. (2011) with their field experience also cited that *C. lateritius* “… has an often excentrical, sometimes laterally compressed, short to long, more or less yellow stipe that can remain white at the base but is concolorous with the cap higher up, and it has an almost smooth to clearly veined often slightly pinkish tinted hymenophore (the senior author has never seen absolutely smooth specimens)...”. Based on our revision of the epitype of *C. lateritius* (Buyck 07.025 kept at PC, designated by Buyck and Hofstetter 2011), it microscopically differs from *C. veraecrucis* by the basidiospores shape (ellipsoid to slightly phaseoliform) and the terminal hyphae of the pileipellis, which are (19–) 21–60 (–70) × 5–11 µm, cylindrical to subclavate, tending to be wider than those of *C. veraecrucis* (Fig. 7).

The Asian *Cantharellus hiananensis* N.K. Zeng, Zhi Q. Liang & S. Jiang, appears related also to *C. veraecrucis*, but according to data by An et al. (2017), it differs from the Mexican species by its smaller basidiome size (pileus 25–55 mm diam., stipe 30–55 × 8–10 mm), paler hymenophore (cream to yellowish white), stipe usually hollow covered with tiny, yellow to pale yellowish brown scales, smaller, subcylindrical basidiospores [6–7.09–8 (–9) × (4–) 4.5–4.84–5 (–5.5) µm], and smaller basidia (50–70 × 7–10 µm), (4–) 5 (–6) -spored and pileipellis terminal hyphae 23–82 × 3–8 mm, narrowly clavate or subcylindrical, sometimes subfusiform, with obtuse apex.

*Cantharellus veraecrucis* represents a wild edible mushroom that is harvested for consumption and commercialization during the rainy season, in the study site and surroundings; it is known as “Oak mushroom”. After our systematic multiyear sampling of basidiomes in the forests studied, we could observe that *C. veraecrucis* is a frequent chanterelle, and shares the same habit preferences as *C. violaceovinosus*, recently described from the same region (Herrera et al. 2018).

##### Cantharellus parvoflavus

Taxon classificationFungiScleractiniaFungiidae

M. Herrera, Bandala & Montoya sp. nov.

F88E8CBA-A1CE-56A3-AFF4-AE3DFE6B75D1

838106

Figs 2c, d , 4

###### Holotype.

Mexico. Veracruz: Municipality of Alto Lucero, NE Mesa de Venticuatro, 450–500 m a.s.l. gregarious, on ground, under *Quercus oleoides* Schltdl. & Cham., 2 Oct 2017, Montoya 5423 (XAL).

###### Diagnosis.

Differing from other related Cantharellus species (subgenus Parvocantharellus) by the pileus surface with appressed fibrils at center, broadly ellipsoid basidiospores 6–9 (–9.5) × 4.5–5 µm [*Q*–= 1.52–1.57 (n=3)], pileipellis terminal hyphae (23–) 25–75 (–80) µm × (3.5–) 4–8 µm, mostly cylindrical, often subclaviform, subventricose or somewhat narrowly utriform.

###### Gene sequences ex-holotype.

nLSUMT371337; *tef*-1α MT449706.

###### Etymology.

Referring to a small, yellow chanterelle; from *parvus* (Lat.): small and *flavus* (Lat.): yellow

###### Description.

***Pileus*** 6–26 mm diam, subhemispheric in young, becoming convex to plane convex and centrally depressed, some finally irregularly infundibuliform; margin incurved when young, becoming inflexed to somewhat straight, undulate or irregular or more or less crenate, not or obscurely translucid striate; surface dry, with appressed fibrils at center when young, glabrous at remaining areas, with waxy appearance, bright yellow-orange (5A5–A8) with tiny white to light yellow scales in the center when young, paler at edge when young. ***Hymenophore*** decurrent or shortly decurrent, with gill-like folds up to 2 mm deep, subdistant to more frequently distant, at times forked, moderately thick with margin entire or often irregular or eroded, frequently intervenose, some specimens (especially towards the stipe) with irregular low and sinuous veins, often with lower irregular anastomosis among the folds, in some specimens the anastomosis occur practically in the whole hymenophore, while in others only at some areas, especially at pileus margin, with some short lamellulae-like folds, concolorous with the pileus. ***Stipe*** (10–) 15–42 × 2–6 mm, broadened towards the apex, somewhat fused, compressed at times or furrowed, solid but soon fistulous to hollow, glabrous, concolorous with pileus. ***Context*** fleshy, concolorous with pileus or somewhat paler, with waxy appearance, odor mild, agreeable; taste mild, agreeable.

***Basidiospores*** 6–9 (–9.5) × 4.5–5 µm [*X*– = 7.6–7.8 × 4.9–5 µm, *Q*– = 1.52–1.57 (n = 3)], broadly ellipsoid, smooth, thin-walled, hyaline, inamyloid, with granular contents or refractive droplets. ***Basidia*** 50–83 (–89) × (6–) 7–10 µm, narrowly clavate to subcylindrical, with 2–5 sterigmata, thin-walled, hyaline; subhymenium composed of cylindrical hyphae 4–6 µm diam. ***Cystidia*** absent. ***Pileipellis*** composed of intermingled hyphae of 4–7 µm diam, cylindrical, hyaline, yellowish in group, terminal hyphae (23–) 25– 75 (–80) × (3.5–) 4–8 µm, mostly cylindrical, often subclaviform, subventricose or somewhat narrowly utriform, moderately straight to flexuous, inamyloid, thick-walled (<1 µm thick), smooth, hyaline. ***Pileus trama*** composed of cylindrical to inflated hyphae, 4–7 µm diam, slightly thick-walled (<1 µm thick), hyaline, some with weakly refringent contents. ***Hymenophoral trama*** composed of hyphae 4–5 µm diam, thin-walled, some with weakly refringent contents. Clamp connections present in all tissues.

**Figure 4.**
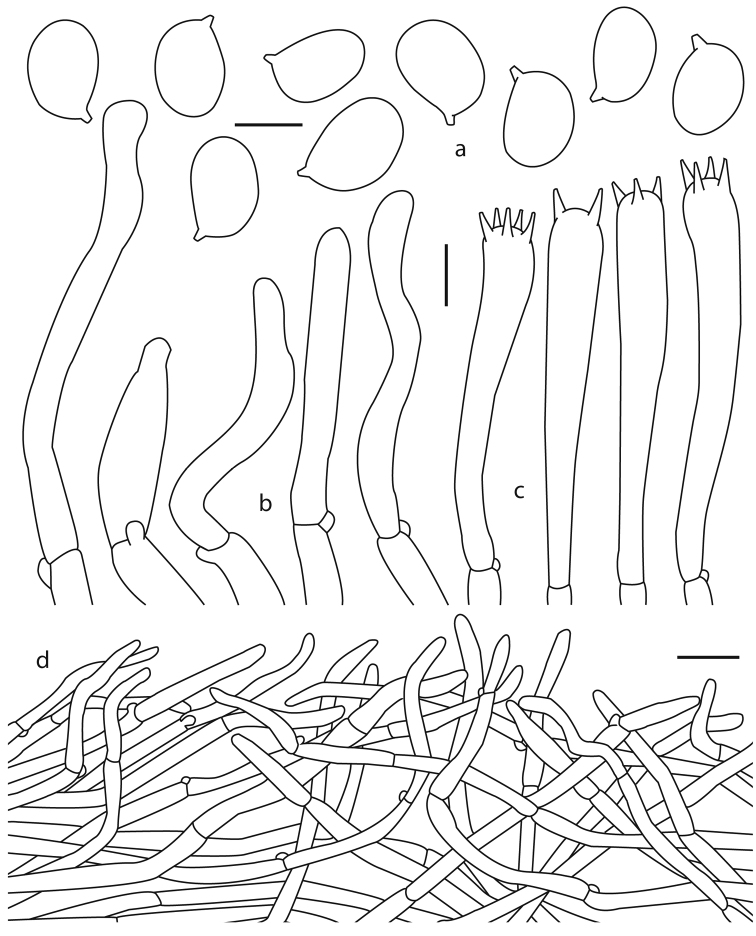
*Cantharellus parvoflavus* (Montoya 5423, holotype) **a** basidiospores **b** Terminal elements of the pileipellis **c** basidia **d** longitudinal section of pileipellis. Scale bars: 5 μm (**a**); 10 μm (**b, c**); 25 μm (**d**).

###### Habitat.

Solitary to gregarious, rare in the study area, on soil, in tropical oak forest, under *Quercus oleoides*, September-October, known in the coastal plain of central Veracruz State, east coast of Mexico.

###### Specimens examined.

Mexico. Veracruz, Municipality of Alto Lucero, NE Mesa de Venticuatro, 392–433 m a.s.l., 27 Sep 2016, Herrera 204; 20 Oct 2017, Herrera 229 (all at XAL).

###### Remarks.

The phylogenetic analysis supports (with high values of bootstrap and Bayesian posterior probabilities 100/1) the distinction of *Cantharellus parvoflavus* as a new species, sister to *C. appalachiensis* from USA. This latter species, besides their basidiomes being somewhat larger [pileus 10–50 mm/stipe 15–75 × 3–10 (–13) mm], are not distinctly yellow, only dingy yellow, usually dull brown, pale or yellowish-brown at margin, darker to brown on disc (Petersen and Ryvarden 1971; Bigelow 1978). Moreover, *C. appalachiensis* has wider broadly-ellipsoid basidiospores [(6.6–) 7.4–8.2 (–8.9) × (4.4–) 4.8–5.6 (–5.9) µm or (6–) 7.5–9 (–10.5) × (4–) 4.5–5.5 (–6) µm] and wider pileipellis hyphae (3–14.5 µm diam. or 9–14 µm diam.) (Petersen and Ryvarden 1971; Bigelow 1978).

*Cantharellus parvoflavus* is similar to yellow forms of *C. minor*, because they have a hygrophoroid appearance, but this latter is usually bright yellow orange to orange, fading to pale orange-buff or pale orange, with glabrous pileus surface, bigger, ellipsoid, slightly phaseoliform basidiospores (6–) 7.5–10 (–11.5) × (4–) 4.5–6 (–6.5) µm and pileipellis terminal elements subcylindrical to subventricose (Bigelow 1978; Buyck et al. 2010). *Cantharellus romagnesianus* is close to *C. parvoflavus* but it develops grey-brown colors in the pileus, its hymenophore has forked veins, often spaced, larger basidiospores [(8–) 9–11.5 (–12.5) × 4–6 (–6.5), Q = 1.71–2.28] and with different shape (Olariaga et al. 2017).

#### New record of *Cantharellus tabernensis* in Mexico

##### Cantharellus tabernensis

Taxon classificationFungiScleractiniaFungiidae

Feib. & Cibula, Mycologia 88: 299 (1996)

8752B33B-2B07-5870-BA23-D7FCBAAA9A81

Figs 2e, f , 5

###### Description.

***Pileus*** 10–30 mm diam, hemispheric to convex, becoming broadly conical to plane convex and faintly depressed in the disc, margin incurved when young, somewhat inflexed to straight with age or somewhat reflexed, not striate, not or faintly undulate or crenulate; hygrophanous, with dull appearance, some with greyish appressed fibrils at center and smooth at the margin when young, smooth to glabrescent with age; light yellow (2.5Y 8/6–8/8, 4A5). ***Hymenophore*** decurrent or shortly decurrent, with gills up to 3 mm deep, subdistant to more frequently distant, continuous, or forked at different levels, moderately thick; margin entire, at times with irregular anastomosis among folds, with short lamellulae-like folds; yellow to egg yellow (10YR 8/8) brighter than the pileus. ***Stipe*** (15–) 19–40 × 2–6 mm, central or at times slightly eccentric, equal, occasionally somewhat applanate, at times slightly fused or broader at base, solid to hollow, often furrowed especially below, hygrophanous, surface smooth, concolorous with the pileus; mycelium whitish to pale yellowish. ***Context*** 1–3 mm thick cream color to yellowish, odor mild, agreeable; taste mild, agreeable.

***Basidiospores*** 6.5–8.5 × 4.5–5 µm [*X*– = 7.32–7.34 × 4.8–4.9 µm, *X*– = 1.49–1.52, (n = 2)], ellipsoid, smooth, thin-walled, hyaline, inamyloid, with granular contents or refractive droplets. ***Basidia*** (53–) 56–87 (–99) × 6–10 µm, narrowly clavate to subcylindrical, with 2–4 sterigmata, thin-walled, hyaline; subhymenium composed of cylindrical hyphae 3–5 µm diam. ***Cystidia*** absent. ***Pileipellis*** a cutis composed of hyphae 5–8 µm diam, intermingled in a compact arrangement, cylindrical, hyaline, inamyloid, with terminal hyphae cylindrical to somewhat subclavate, 62–75 × 6–10 µm, slightly thick-walled (<1 µm thick), smooth, hyaline, inamyloid, usually abundant. ***Pileus trama*** composed of cylindrical hyphae, 3–8 µm diam, slightly thick-walled (<1 µm thick), hyaline. ***Hymenophoral trama*** composed of hyphae 3–6 µm diam, thin-walled. Clamp connections present in all tissues.

**Figure 5.**
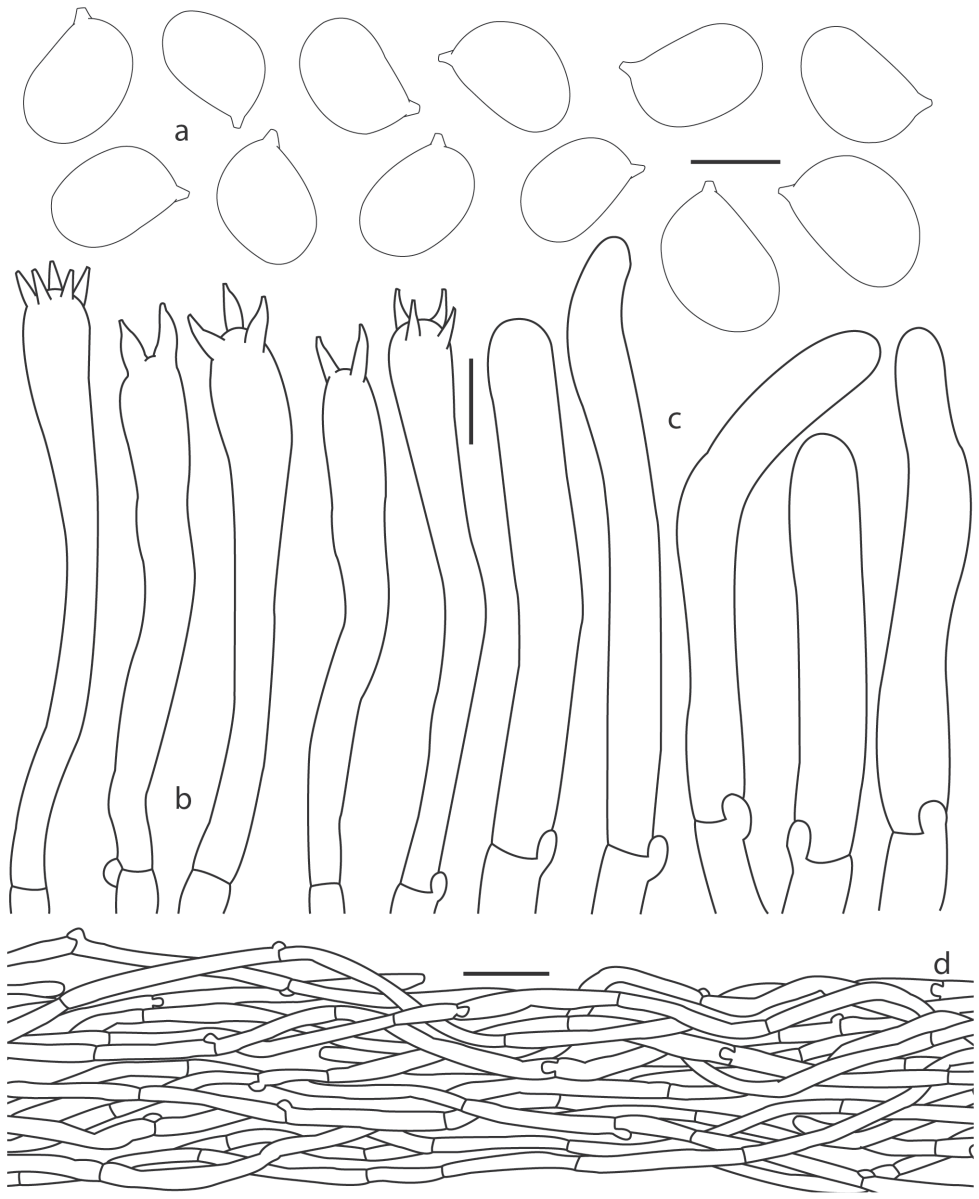
*Cantharellus tabernensis* (Herrera 131) **a** basidiospores **b** basidia **c** Terminal elements of the pileipellis **d** longitudinal section of pileipellis. Scale bars: 5 μm (**a**); 10 μm (**b, c**); 25 μm (**d**).

###### Habitat.

Solitary to gregarious, rare in the study area, on soil, in tropical oak forest, under *Quercus oleoides* and *Q. sapotifolia*, fruiting in June at the coastal plain of central Veracruz State, east coast of Mexico.

###### Specimens examined.

Mexico. Veracruz, Municipality of Zentla, Road Puentecilla-La Piña, 837 m a.s.l.,11 Jun 2015, Herrera 120, 121;10 Sep 2015, Herrera 131 (all at XAL).

###### Remarks.

In our phylogeny Mexican sequences of specimens Herrera 120 and 121 clustered (Fig. 1) with high values of Bootstrap and Bayesian posterior probabilities (96/0.99) with a sequence of the type specimen of *Cantharellus tabernensis* from U.S.A., produced by Buyck et al. (2014). The morphological description provided above includes both mentioned specimens, and in fact, in the most relevant characters, those specimens agree with the species. It should be mentioned, however, that the following features recorded in the description provided by Feibelman et al. (1996) were not observed in the Mexican material: pileus mat felted overall, often umbilicate, sometimes perforated, basidia 4–5–6 -spored and dark plasmatic pigment confined to clavate terminal cells of the surface hyphae at disc.

The record presented here of *C. tabernensis*, in its turn provides additional information on the species distribution. It is known from the mixed pine and hardwood forests, usually near *Pinus elliotii* Engelm., at the Gulf coastal plain in Texas, Mississippi, and Louisiana states in USA (Feibelman et al. 1996), and now *C. tabernensis* is known also in the tropical *Quercus* forest from Veracruz, in the coastal plain of Veracruz state in the Gulf of Mexico.

#### Proposal of a new name for the replacement of *Craterellus confluens*

Derived from the fact that *Craterellus confluens* was described by Berkeley (1867) from the Orizaba region in Veracruz (Mexico), later records of yellow chantherelles occurring in the Zentla region (north of Orizaba) were referred to in the past by Guzmán and Sampieri (1984) as “*Cantharellus odoratus*” following Corner (1966). This latter author introduced that *C. lateritius* and *Cr. confluens* were the same as Schweinitz (1822) described as *Merulius odoratus*. Burt (1914) mentioned particularly the macroscopic resemblance among the *Cr. confluens* isotype specimen in Schweinitz herbarium and the specimens of *C. odoratus* that he studied, thus he synonymized the former and pointed out that “…the type of *Cr. confluens* has the hymenium rugose-wrinkled, as is often the case in specimens of *C. odoratus*; its habit, dimensions, structure, coloration, and spores are quite those of *C. odoratus*…”. In the molecular phylogeny here generated (Fig. 1) *Cr. confluens* holotype specimen (Botteri 6, kept at K) is supported with high values of bootstrap and Bayesian posterior probabilities sister to *C. veraecrucis* here described (above), and both are closely related with *C. lateritius* (including a sequence of the type) and *C. flavolateritius*. Petersen (1979) after type studies considered indeed, separately *C. lateritius*, *Cr. odoratus* (Schwein.) Fr. and *Cr. confluens*, being a combination of characters such as clamps (present or not), basidiome colors and the leathery, funnel-shaped basidiocarps (with a hollow stipe), among other features, considered in the distinction of such taxa. Molecular studies have also shown that Schweinitz’s species belongs to *Craterellus* (i.e. *Cr. odoratus*) (Feibelman et al. 1997; Dahlman et al. 2000) and now, our analysis confirms (Fig. 1) that *Cr. confluens* holotype specimen belongs to *Cantharellus*, among the group of yellow species around *C. lateritius*. Buyck and Hofstetter (2011) suggested “…to refrain from using the name *C. confluens* any longer…”, but rather a new specific name in *Cantharellus* is required for such taxon because in *Cantharellus* the specific name is preoccupied by *C. confluens* (Schwein.) Schwein. 1834, i.e. *Merulius confluens* Schwein. 1822, a meruliod species (Burt 1917) member of *Byssomerulius* (Ginns 1975; Zmitrovich et al. 2006). Possibly *Cr. confluens* exhibits a rare occurrence in the site that we explored in the Zentla region or it has a more restricted occurrence in some other ecosystem, near or around the city of Orizaba, Veracruz. Considering the features of the fruitbodies (“…stem divided…”) mentioned by Berkeley (1867) in the diagnosis, we propose to replace the name as follows:

##### Cantharellus furcatus

Taxon classificationFungiScleractiniaFungiidae

Bandala, Montoya & Ramos nom. nov.

234CFE08-12A4-5D7D-8EB7-A3EABC316ACB

838107

Bas. Craterellus confluens Berk. & M.A. Curtis, J. Linn. Soc., Bot. 9: 423 (1867).
Syn. Cantharellus confluens (Berk. & M.A. Curtis) R.H. Petersen, Sydowia 32: 201 (1979) nom. illeg.
Non Cantharellus confluens (Schwein.) Schwein., Trans. Am. Phil. Soc., New Series 4: 153 (1834).
= Merulius confluens Schwein., Schr. Nat. Ges. Leipzig 1: 92 (66 in reprint) (1822).
= Byssomerulius corium (Pers.: Fr.) Parmasto, Eesti NSV Tead. Akad. Toim., ser Biol. 16: 383 (1967).

###### Holotype.

Mexico. Veracruz, Orizaba. Botteri 6 [ex herb. M.J. Berkeley] KM 173247 (K).

###### Gene sequences ex-holotype.

nLSUMT371345.

###### Etymology.

From *furcatus* (Lat.): forked, referring to a bifurcation developed in the basidiome.

###### Remarks.

Presumably having been separated from the entire collection, the holotype specimen consists of a single unipileate basidiome but the diagnostic feature mentioned by Berkeley (1867) “… stem divided above into numerous pilei…”, a feature practically not observed in close related species (*C. flavolateritius*, *C. lateritius*, *C. veraecrucis*) is present, as noted and depicted by Burt (1914), in the isotype collection at Farlow Herbarium (https://huh.harvard.edu/pages/farlow-herbarium-fh), and it is well-depicted and described for collections from SE USA studied by Petersen (1979). The particularity of producing multipileate basidiomes and/or with fused stipes, in combination with the smooth pileus surface, pileus and hymenophore predominantly orange colored (*aurantiacus* in the diagnosis) hymenophore rugulose, irregularly forking and anastomosing, rarely smooth, with yellow stipe and lacking pinkish shades (Petersen 1979), are the distinctive macroscopic features of *C. furcatus*.

**Figure 6.**
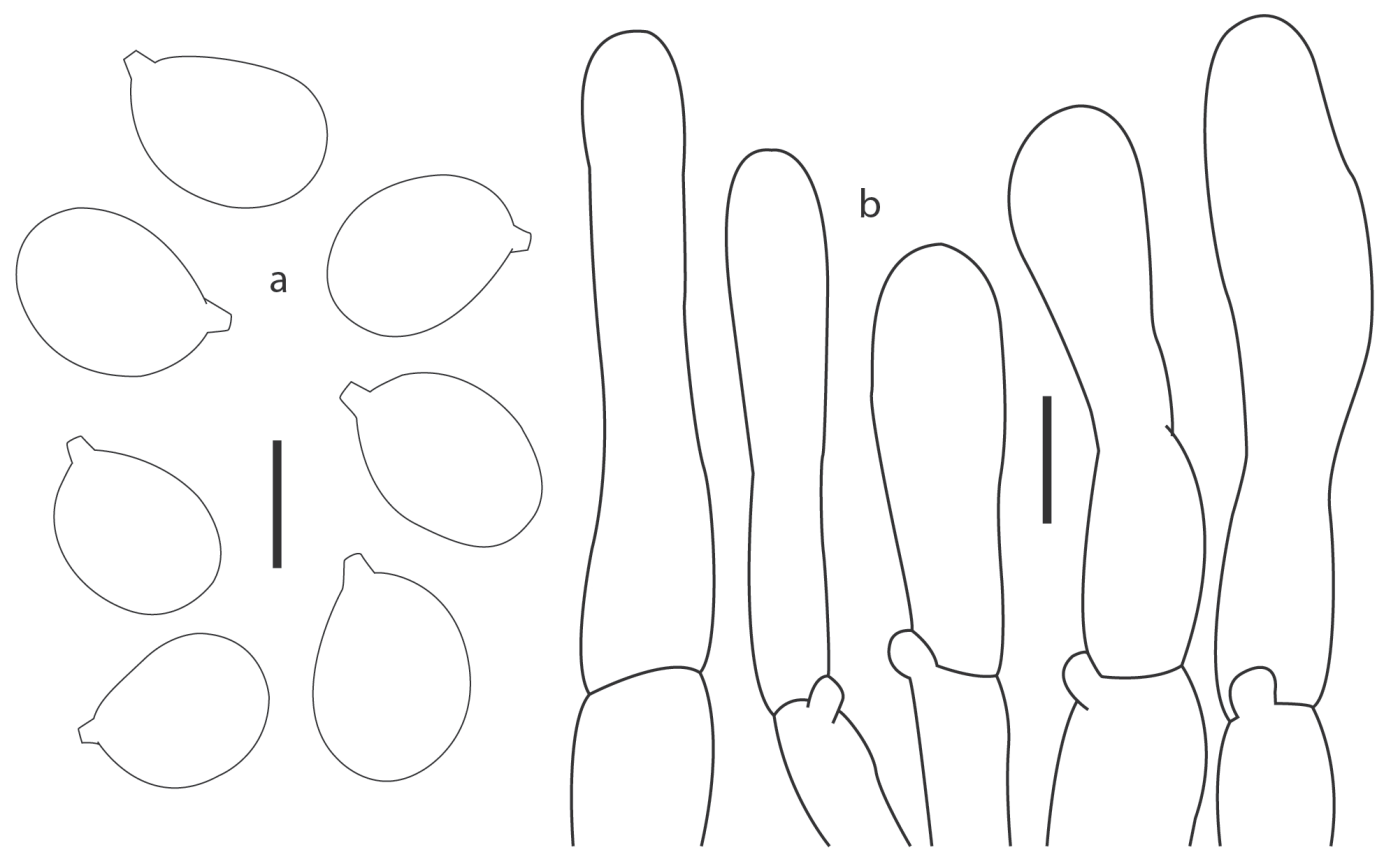
*Cantharellus furcatus* (Botteri 6, holotype of *Craterellus confluens*) **a** basidiospores **b** terminal elements of the pileipellis. Scale bars: 5 μm (**a**); 10 μm (**b**).

The holotype specimen Botteri 6 (at K) of *Cr. confluens* was preserved in such a poor condition that it does not allow a proper rehydration of the tissues. The microscopic features recovered were: basidiospores of 7.5–8.5 × 5–6 µm (*X*– = 7.8 × 5.3 µm), *Q*– = 1.46, broadly ellipsoid to ellipsoid, some subglobose, somewhat flattened adaxially, smooth, hyaline, thin-walled, inamyloid. Pileipellis a cutis composed of cylindrical hyphae 5–7 µm diam, compactly arranged, hyaline, yellowish colored in group; terminal hyphae 36–57 × 8–12 µm, clavate to broadly clavate, scattered, smooth, hyaline, inamyloid, thin to thick-walled (<1 µm thick). Clamp connections present (Fig. 6). In the holotype Petersen (1979) registered basidiospores of 6.7–8.9 × 4.8–5.9 µm, *Q*– = 1.29–1.54 and of 7–10 × 5–6.3 µm, while in the isotype collection there is an annotation made in 1980 by Dr. H.E. Bigelow, describing basidiospores: 8–10 × 5.5–6.5 µm, ellipsoid or broadly ellipsoid or subglobose, smooth, inamyloid, basidia mostly collapsed, ± 41–52 × 6–7.5 µm, pileus with hyphae 4–10 µm diam, clamped, pigment apparently intracellular (https://huh.harvard.edu/pages/farlow-herbarium-fh).

**Figure 7.**
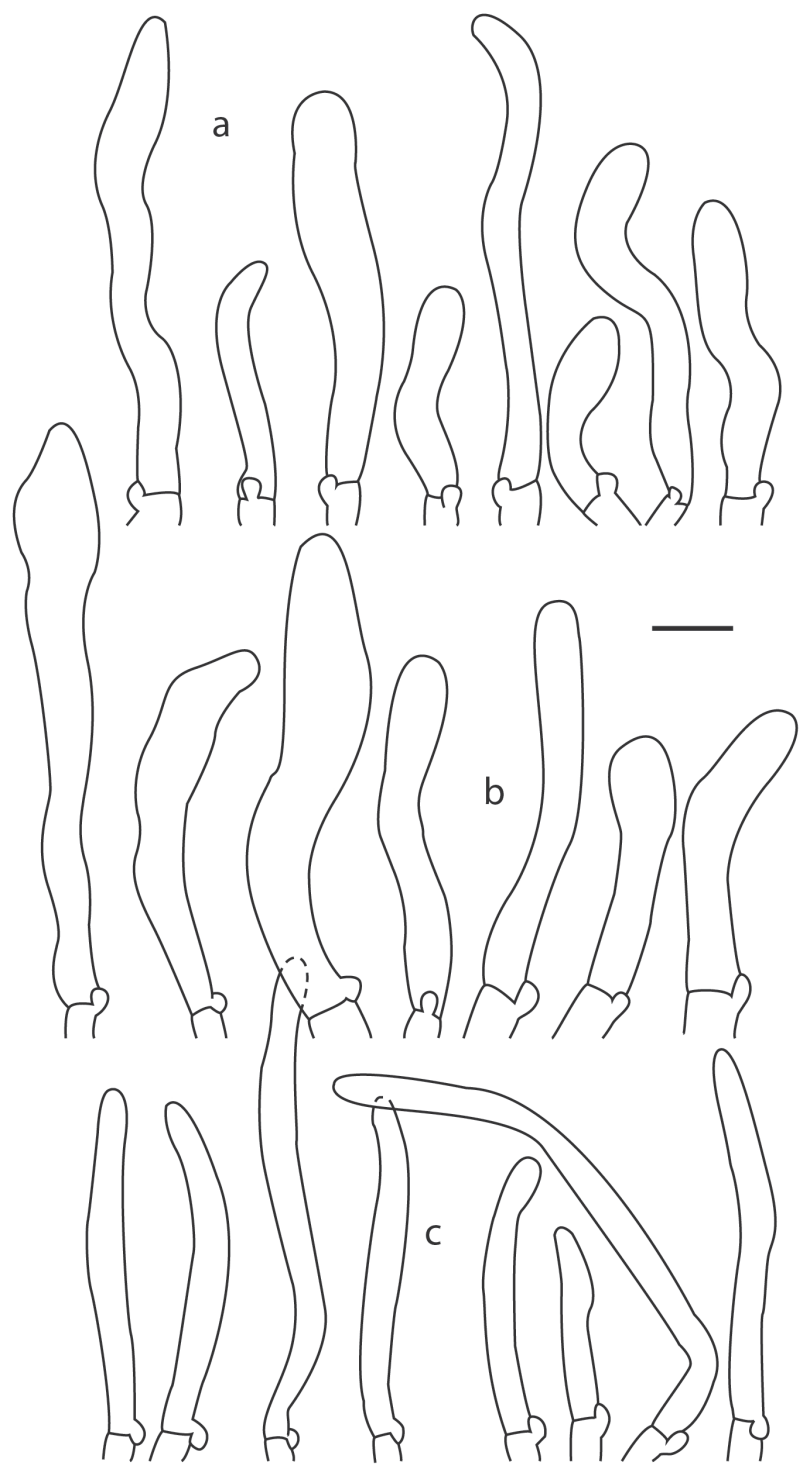
Terminal elements of the pileipellis of *Cantharellus* species **a, b***C. lateritius* (**a** Buyck 05.058 **b** Buyck 07.025 epitype) **c***C. veraecrucis* (Bandala 4505, holotype). Scale bar: 10 μm.

## Supplementary Material

XML Treatment for Cantharellus veraecrucis

XML Treatment for Cantharellus parvoflavus

XML Treatment for Cantharellus tabernensis

XML Treatment for Cantharellus furcatus
